# Patient-reported adherence to statin therapy, barriers to adherence, and perceptions of cardiovascular risk

**DOI:** 10.1371/journal.pone.0191817

**Published:** 2018-02-08

**Authors:** Vicki Fung, Ilana Graetz, Mary Reed, Marc G. Jaffe

**Affiliations:** 1 Mongan Institute Health Policy Center, Massachusetts General Hospital, Boston, Massachusetts, United States of America; 2 Department of Medicine, Harvard Medical School, Boston, Massachusetts, United States of America; 3 Department of Preventive Medicine, University of Tennessee Health Science Center, Memphis, Tennessee, United States of America; 4 Division of Research, Kaiser Permanente Northern California, Oakland, California, United States of America; 5 Resolve to Save Lives, New York, New York, United States of America; 6 Department of Endocrinology, Kaiser Permanente South San Francisco Medical Center, San Francisco, California, United States of America; University of Tampere, FINLAND

## Abstract

**Background:**

Patient reports of their adherence behaviors, concerns about statins, and perceptions of atherosclerotic cardiovascular disease (ASCVD) risk could inform approaches for improving adherence to statin therapy. We examined these factors and their associations with adherence.

**Methods:**

We conducted telephone interviews among a stratified random sample of adults receiving statins within an integrated delivery system (N = 730, 81% response rate) in 2010. We sampled equal numbers of individuals in three clinical risk categories: those with 1) coronary artery disease; 2) diabetes or other ASCVD diagnosis; and 3) no diabetes or ASCVD diagnoses. We assessed 15 potential concerns about and barriers to taking statins, and perceived risk of having a heart attack in the next 10 years (0–10 scale). We calculated the proportion of days covered (PDC) by statins in the last 12 months using dispensing data and used multivariate logistic regression to examine the characteristics associated with non-adherence (PDC<80%). Analyses were weighted for sampling proportions.

**Results:**

Sixty-one percent of patients with PDC<50% reported not filling a new prescription, splitting or skipping statins, or stopping refilling statins in the last 12 months vs. 15% of those with PDC≥80% (p<0.05). The most commonly reported concerns about statins were preferring to lower cholesterol with lifestyle changes (66%), disliking medications in general (59%), and liver or kidney problems (31%); having trouble remembering to take statins (9%) was the most common reason for taking less than prescribed. In multivariate analyses, clinical risk categories were not significantly associated with odds of non-adherence; however, those with higher perceived risk of heart attack were less likely to be non-adherent.

**Conclusions:**

Patient-reported medication-taking behaviors were correlated with statin PDC and those with lower perceived cardiovascular risk were less likely to be adherent. These findings highlight the importance of eliciting from and educating patients on their adherence behaviors and ASCVD risks.

## Introduction

The American College of Cardiology and the American Heart Association (ACC-AHA) released updated guidelines for the management of cholesterol in 2013 expanding the number of individuals for whom statins are recommended for primary prevention.[1] These guidelines introduced an ASCVD risk threshold (7.5% or greater over 10 years) above which statin therapy is recommended for primary prevention.[2] Estimates suggest that these recommendations could increase the number of Americans eligible for statin treatment by about 14 million to 56 million adults overall.[3] Among the newly eligible are a large number of patients without clinical atherosclerotic cardiovascular disease (ASCVD), but with 10-year CVD risks of 7.5% or greater, who are recommended to receive statins for primary prevention.

Expanded use of statins could prevent a substantial number of cardiovascular events.[3] Sub-optimal adherence to statins, however, reduces the possibility of realizing this reduction.[4–6] For example, a recent study found that about one-third of non-elderly adults receiving statins were non-adherent over a 12-month period based on having medication possession ratios less than 80%.[7] In a previous focus group study among patients who were non-adherent to their statin therapy, we found a wide range of attitudes and perceptions about statins that contribute to poor adherence including inconvenience, lifestyle preferences, and uncertainty about risks and benefits of statins.[8] Less is known, however, about the frequency of these concerns and the extent to which patients actually reduce statin use because of them. A wide-range of approaches to increase statin adherence, including those focused on patient education and support, reminders, and reducing regimen complexity and costs, appear to have significant and positive, but relatively modest effects on adherence.[9–15] Better tailoring interventions to individual patient adherence behaviors, attitudes, and actual or perceived CVD risk could improve their effectiveness.

In this study, we conducted a telephone interview survey in an integrated delivery system to assess patient reports of their statin adherence behavior, their concerns about and barriers to taking statins as prescribed, and perceived CVD risks. We linked these data with health system pharmacy data on dispensed statin prescriptions to compare self-reported adherence behaviors with the proportion of days covered (PDC) by statins. To assess patterns by clinical risk level, we also linked the survey data with health system medical records to identify clinical risk categories of patients who were taking statins for secondary prevention (i.e., those with coronary artery disease), those with a diagnosis of diabetes or other clinical ASCVD without coronary artery disease (CAD), and those receiving statins for primary prevention.

## Methods

### Study design and setting

We conducted a cross sectional telephone survey in 2010 among adult members of Kaiser Permanente Northern California (KPNC) who had been dispensed a statin in 2009. KPNC is an integrated delivery system that provides comprehensive care, including inpatient, outpatient, and pharmacy services, to more than three million members.

At the time of the survey (2010), the KPNC guidelines for adult cholesterol management, consistent with national guidelines, recommended statin therapy for patients with acute coronary syndrome, CAD or ischemic stroke/transient ischemic attack (TIA), diabetes mellitus (DM, age 40 years and older), peripheral arterial disease, abdominal aortic aneurysm, or carotid artery stenosis (>50%). In addition, statins were recommended for patients with Framingham 10-year risk levels>20% or DM (under age 40) with one or more risk factors if they also had LDL-c ≥100 mg/dL; those with DM (under age 40) without risk factors with LDL-c≥130 mg/dL; those with chronic kidney disease (stages 4 and 5) with LDL-c≥100 mg/dL; or those with Framingham 10-year risk between 10–20% and LDL-c≥130 mg/dL. For those with Framingham 10-year risk <10%, statins were recommended for patients with LDL-c≥190 mg/dL; for those with Framingham 10-year risk between 5–9% and family history of premature CAD, statins were recommended for those with LDL-c≥130 mg/dL.

Simvastatin was the recommended first-line treatment within the health system at the time of the survey and was available to members at the generic copayment level. The health system does not accept copay coupons from manufacturers.

### Study population

Our study included a random sample of adult KPNC members, aged 18 years and older with at least one statin dispensed between Jan 1, 2009 and May 31, 2009. We sampled an equal number of individuals in each of three clinical risk categories: 1) Those receiving statins for secondary prevention based on membership in the health system’s CAD registry (e.g., 1+ hospitalization for acute myocardial infarction (AMI) diagnosis, coronary artery bypass grafting (CABG) or percutaneous transluminal coronary angioplasty (PTCA) procedure code); 2) Those receiving statins because they had a diagnosis of diabetes (DM) or other ASCVD (i.e., diagnosis of DM, stroke or transient ischemic attack, peripheral arterial disease, or abdominal aortic aneurysm at baseline), and 3) those receiving statins for primary prevention (i.e., those with no ASCVD or diabetes diagnosis).

To increase our ability to examine factors associated with poor adherence we also over-sampled individuals with lower statin adherence levels within each clinical risk category. Specifically, we calculated the proportion of days covered by statins in the 12-month period from Jun 1, 2009-May 31, 2010 using pharmacy dispensing data and sampled equal numbers of individuals with high PDC (> = 80%), medium PDC (50–79%), and low PDC (<50%). We applied survey design weights based on the inverse of the sampling fraction to account for the stratified sampling. All analyses are weighted by these proportions to represent the overall target population of adults receiving statins in the health system.

### Recruitment

Starting in July 2010, potential participants received a mailed study letter, questionnaire, and reply postcard. They could decline participation via postcard or telephone, or return the questionnaire by mail. Phone interviewers contacted the remaining potential participants. If the respondent was reached by phone, the interviewers explained the study and verbal consent was obtained prior to starting the interview. The Kaiser Permanente Northern California institutional review board approved the study and waived the requirement of written informed consent from study participant. All participants received a $5 coffee gift card.

The response rate was 81% with 730 completed interviews. The sample size for this survey was based on prior survey studies we have conducted in similar patient populations examining drug adherence.[16–18] Participants were ineligible if they could not complete the interview due to language barriers, cognitive impairment, or illness (N = 139); if they could not be reached due to incorrect contact information, after 15 or more attempts, or if they were non-community dwelling or no longer KPNC members (N = 189); or if they reported that they had not been prescribed a statin in the previous 12 months in an initial interview screening question (N = 72). Participants were similar to non-participants with respect to age and gender. In our final analyses, we also excluded individuals who reported on the survey that they had filled any statin prescriptions outside of KPNC in the last 12 months (N = 31) because the PDC calculation was based on health system pharmacy data that does not include out-of-system fills.

### Survey measures

#### Adherence

To assess self-reported adherence to statin therapy, we asked participants if they engaged in any of the following behaviors in the last twelve months: (1) not filling a prescription for a new statin medication; (2) splitting or skipping statin pills without their doctor’s advice; and (3) stopped refilling a statin prescription all together without their doctor’s advice.[16–20] We compared participants’ self-reported adherence behaviors in the last 12 months and their statin PDC over the same 12-month period (<50%, 50–79%, and 80–100%) based on pharmacy data.

#### Barriers to adherence

To assess participant’s concerns and barriers to statin drug therapy adherence, we asked about 15 specific barriers, which we grouped into four categories: (1) concerns about the need for or benefits of statins (i.e., unsure about why you were prescribed a statin, unsure if statins lower your cholesterol or risk of heart disease); (2) logistical barriers (i.e., trouble paying for statins, inconvenient to obtain, trouble remembering to take statins, too difficult or complicated to take statins); (3) concerns about side-effects or adverse effects (i.e., believe the risks of taking statins outweigh the benefits, concern about muscle problems, concern about liver or kidney problems, concern about other side effects, concern about harmful interactions with other medications, concern about negative long-term health effects); and (4) lifestyle preferences (i.e., concern about having to take statins for the rest of your life, dislike taking medications in general, prefer to lower cholesterol with lifestyle changes such as exercise and diet). For each specific barrier and aggregate category, we examined the percent of participants who reported the concern, and if so, whether they reported taking less of their statin medication than prescribed in the last 12 months because of the concern.

#### Perceived cardiovascular risk

To assess participants’ perceived CVD risk, we asked them to rate how likely they thought it was that they would have a heart attack in the next 10 years. We used a 0 to 10 scale where 0 indicated they thought there was absolutely no chance, 5 indicated there was an equal chance of it happening or not, and 10 indicated they thought it was absolutely sure to happen.[21, 22]

#### Analysis

We examined how patients’ risk characteristics were associated with poor adherence (PDC<80%) using multivariate logistic regression including the participant’s clinical risk category (CAD, DM/other ASCVD, or neither) and their perceived CVD risk, which we categorized as low (0–2), moderate (3–6), or high (7–10). In sensitivity analyses, we classified perceived CVD risk using different cutoffs and examined the associations between clinical risk categories and adherence without adjusting for perceived risk level; results were similar. We also controlled for individuals’ characteristics available from the administrative data, including age, gender, and whether the participant had a primary care provider (PCP), and characteristics obtained from the survey, including race/ethnicity, education level, marital status, annual household income in 2009, number of medications prescribed in the last twelve months, self-reported health status, and types of concerns about statins.

## Results

### Study population characteristics

**Fig 1:**
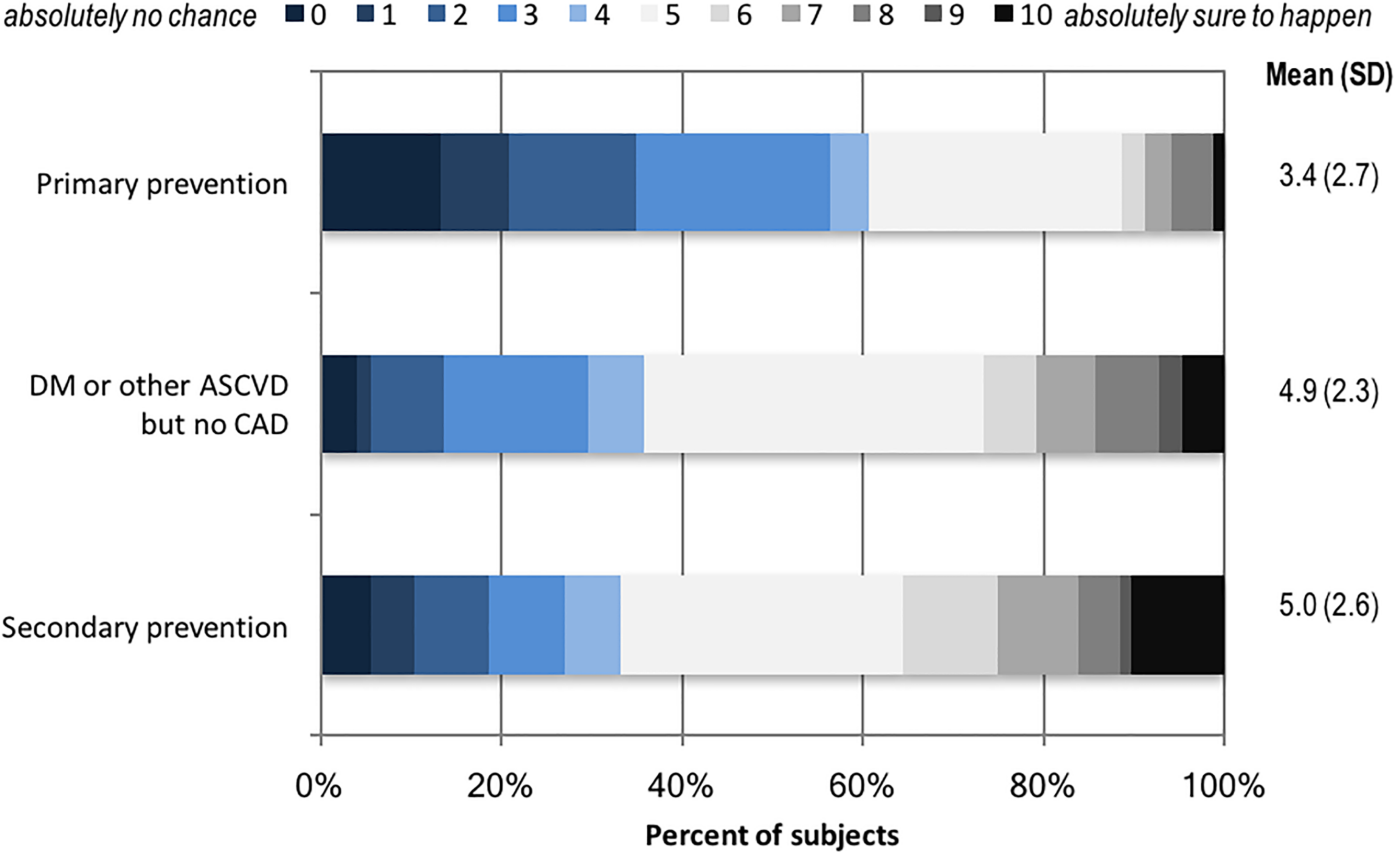
Patients’ perceived risk of heart attack in the next 10 years by clinical risk category. Note: Based on patients’ rating of their perceived risk of heart attack in next 10 years: 0 (absolutely no chance) to 10 (absolutely sure to happen). Weighted for sampling proportions.

Overall, 47.9% of the study population were female and 53.6% were age 65 or older, and 76.9% had a 12-month statin PDC>80% (Table 1). Over half (52.0%) were receiving statins for primary prevention, compared with 17.8% with CAD, and 30.2% with DM or other ASCVD. On a 10-point scale, 25.5% of participants rated their risk of having a heart attack in the next 10 years to be low (0–2), 59.1% moderate (3–6), and 15.4% high (7–10). These perceived risks differed across the clinical risk categories: e.g., 8.9% of those receiving statins for primary prevention rated their risk as high (7–10), compared with 25.1% of those with CAD, and 20.9% of those with DM or other ASCVD (p<0.05, Fig 1).

**Table 1:** Study population characteristics.

	Percent of participants
Gender: Female (vs. male)	47.9%
Age: <50	4.9%
50–64	41.5%
65+	53.6%
Self-Reported Race/Ethnicity: Non-white (vs. white)	33.9%
Education: High school of less (vs. some college or more)	28.6%
Married	65.6%
2009 Household Income: <$40,000	31.6%
Self-reported Health Status: Fair, poor, or very poor (vs. good/excellent)	25.8%
Statin PDC in prior 12 months: <50%	7.6%
50–79%	15.6%
80%+	76.9%
Clinical risk category: Primary prevention (No CAD, DM or other ASCVD)	52.0%
DM or other ASCVD	30.2%
Secondary Prevention (CAD)	17.8%
Self-perceived risk of heart attack in next 10 years: 0–2	25.5%
3–6	59.1%
7–10	15.4%

Note: ASCVD = Atherosclerotic cardiovascular disease; self-perceived risk of heart attack in next 10 years: 0 (absolutely no chance) to 10 (absolutely sure to happen).

### Self-reported non-adherence

**Fig 2:**
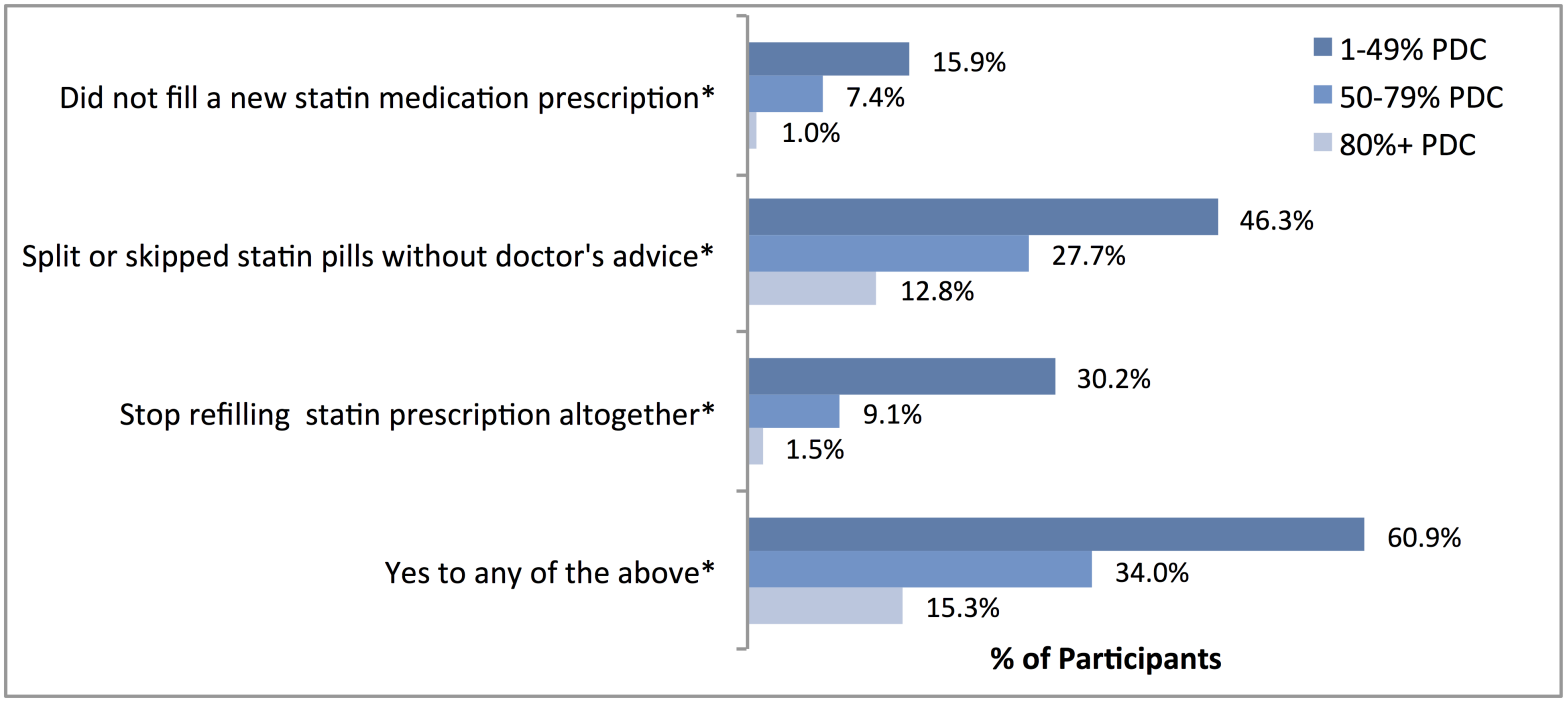
Percent of patients reporting statin non-adherence behaviors in the last 12 months by statin PDC level. Note: Weighted for sampling proportions; *p<0.05.

Fig 2 displays the proportion of participants reporting each of the three non-adherence behaviors by their statin PDC level. Overall, 3.4% reported not filling a new statin prescription, 17.7% split or skipped statin pills without their doctor’s advice, and 4.8% stopped refilling their statin prescription altogether in the last 12 months; 21.8% reported any of these behaviors. We found a strong correlation between reporting each of the three non-adherence behaviors in the last 12 months and PDC level. For example, most participants (60.9%) with low statin PDC (<50%) reported any of the three non-adherence behaviors compared with only 15.3% of those with high statin PDC (>80%, p<0.05).

We examined whether the frequency of these behaviors differed by clinical risk group and did not find significant differences: 19.9% of the primary prevention group reported any of the non-adherence behaviors, compared with 23.8% of those with CAD and 23.9% of those with DM or other ASCVD, respectively. Lastly, 73.7% of those who did not fill a new statin prescription, 28.5% of those who split or skipped pills, and 55.4% of those who stopped refilling statins altogether reported these behaviors to their doctor.

### Barriers to adherence

**Fig 3:**
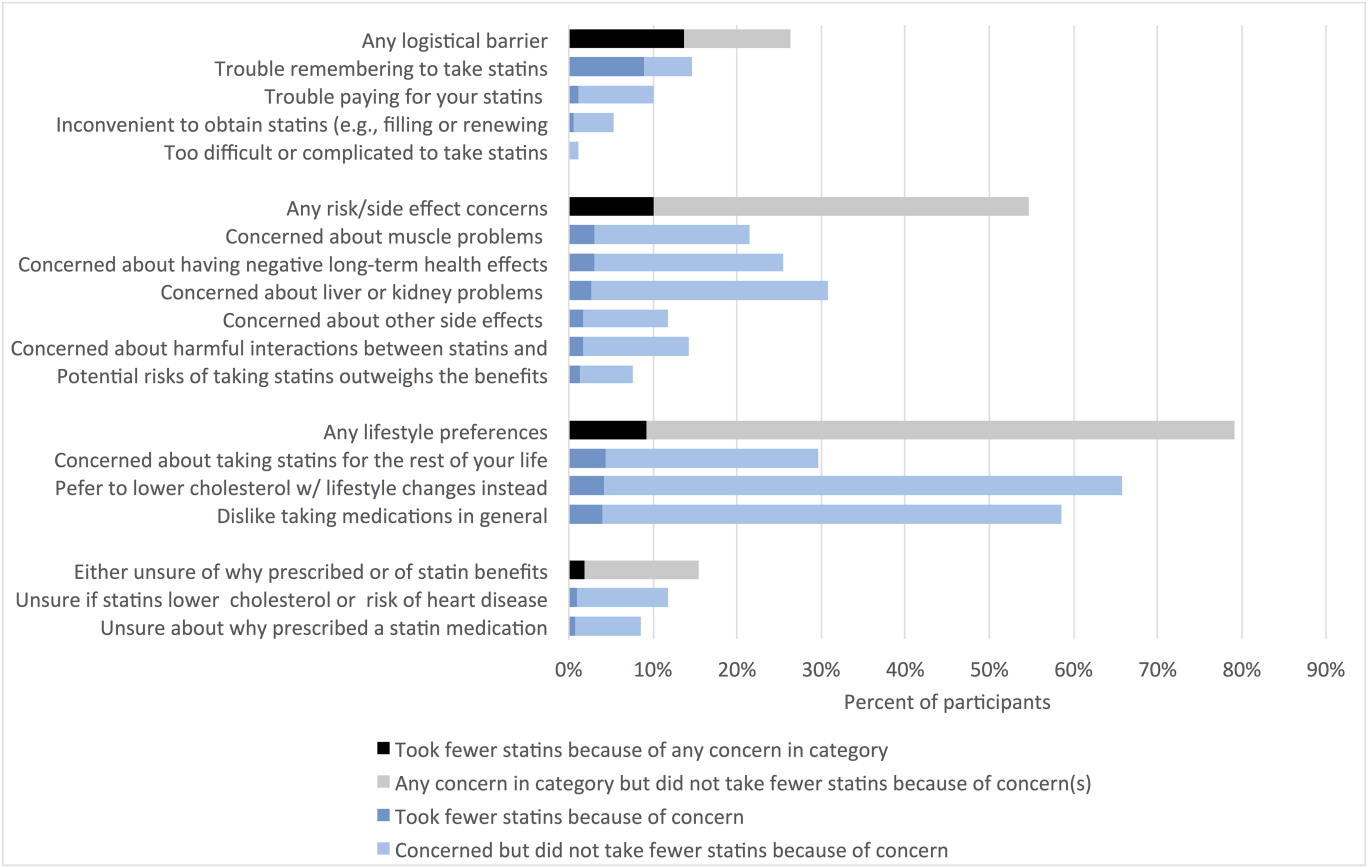
Percent of patients reporting concerns about statins and whether they took fewer statins because of the concern. Weighted for sampling proportions.

Fig 3 presents the total proportion of participants who reported each of the 15 concerns about statins and each of the four aggregate categories of concerns, and the proportion who reported that they took fewer statins because of the concern. The most commonly reported type of concern were about lifestyle preferences (79.1% of participants), including preferring to lower cholesterol with lifestyle changes, such as diet and exercise (65.7%) and dislike of medications in general (58.6%). Only a minority of patients (9.2%), however, reported that they took fewer statins specifically because of these concerns.

In contrast, 26.3% of participants reported any logistical concerns about taking statins, including having trouble remembering to take them (14.6%), or having trouble paying for them (10.1%). Logistical concerns, however, were the most common reason for taking fewer statins than prescribed with 13.7% of participants reporting that they took fewer statins because of any logistical concern; having trouble remembering to take statins being the most common reason.

Over half (54.7%) of participants reported any concerns about the risks or side effects associated with taking statins; 10.1% of participants took fewer statins because of these concerns. Lastly, 15.4% of participants were unsure of whether statins lower their cholesterol or risk of heart disease (11.8%) or why they were prescribed a statin (8.6%). Fewer than 2% took fewer statins because of these concerns.

There were no significant differences across the clinical risk groups or by perceived risk in the frequency of reporting the four types of statin concerns before and after adjusting for other patient characteristics.

### Clinical risk, perceived risk and statin adherence

We assessed the association between clinical risk category, perceived CVD risk, and statin non-adherence (PDC<80%), adjusting for individual characteristics (Table 2). We did not find significant differences in the likelihood of non-adherence across the clinical risk categories. Those with higher perceived CVD risk were less likely to be non-adherent (e.g., OR = 0.51, 95% CI: 0.26–0.996 for those with self-reported risk of heart attack in next 10 years of 7–10 vs. 0–2).

**Table 2:** Association between clinical risk category, perceived risk of heart attack and poor statin adherence.

	Unadjusted % Non-adherent	Adjusted OR	(95% CI)
Clinical risk category: Primary prevention	23.4%	1.0	Ref
DM or other ASCVD but no CAD	19.0%	1.16	(0.88–1.51)
CAD	24.4%	1.13	(0.87–1.48)
Perceived 10-year risk of heart attack: 0–2	27.8%	1.0	Ref
3–6	21.6%	0.66	(0.41–1.04)
7–10	20.1%	0.51*	(0.26–0.996)

*p<0.05

Notes: Non-adherent defined as PDC<80%; clinical risk categories based on health system CAD registry and diagnoses; perceived risk of heart attack in next 10 years based on self-reports on scale from 0 (absolutely no chance) to 10 (absolutely sure to happen). Models adjusted for age, sex, race, marital status, education, income, self-reported health status, having a regular PCP, total number of medications, self-reported type of concerns about statins (unsure why prescribed/benefits, logistical, risk/side effect, lifestyle)

## Discussion

In this study of adults receiving statins within an integrated delivery system, close to one-in-four did not fill adequate statin supply for 80% or more days in the last 12 months. A similar proportion of participants reported non-adherence behaviors in the last 12 months, including not filling or refilling statin prescriptions or skipping pills. Self-reported behaviors were significantly correlated with filled supply, nevertheless, almost 40% of those with low adherence as measured by PDC (<50%) did not report any such behaviors. Our findings agree with previous studies that suggest a moderate correlation between self-reported measures of adherence and electronic measures.[23]

While there is no gold standard for measuring adherence, it was encouraging that most patients who reported primary non-adherence or stopping their statins altogether also reported that they informed their doctors of this change. Those who reported splitting or skipping pills were less likely to report this behavior to their doctor, but these patients were also more likely to have higher levels of adherence, and could have engaged in this behavior only intermittently. Encouraging more direct dialogue between providers and patients about adherence could improve this exchange of information and help identify more patients in need of support to improve adherence.[4, 14]

Among patients receiving statins, the most commonly reported concerns were about lifestyle preferences and the side effects and risks associated with taking statins, but few patients reported actually reducing their use of statins because of these concerns. In contrast, fewer patients reported concerns about forgetting to take their statins, but this was the most common reason for missing doses. These findings are consistent with other studies that have elicited patient-reported reasons for non-adherence as well as the literature on adherence interventions that suggest that those focused more on reminder systems, reducing dosing complexity, and providing monitoring and feedback could be more effective in improving adherence compared with those focused solely on patient education.[24–27]

Other perceptions about statins or ASCVD risk, however, could contribute to forgetfulness if taking statins are not an underlying priority.[9] We found that clinical risk categories as determined by whether patients had established diagnoses of CAD, DM or other ASCVD, vs. no ASCVD diagnoses, were not associated with adherence levels or concerns about statins. However, patients with lower perceived cardiovascular risks were more likely to be non-adherent. While average perceived risk was higher among those with DM or ASCVD versus those without, our study was not powered to examine interactions between clinical risk categories and perceived risk. Nevertheless, our findings suggest that patients’ perceptions of risk are likely to influence medication-taking behaviors and represent another opportunity for provider-patient engagement.[28]

Prior studies have found that actual and perceived risks are often discordant.[22] Thus, clearly communicating ASCVD risk, particularly among patients receiving statins for primary prevention and without elevated cholesterol levels under the revised guidelines, could be important for underscoring the clinical benefits of statins and of improving and maintaining adherence. Interventions that combine behavioral components such as monitoring or reminders, and patient education have been found to be effective in improving adherence.[25] In our study population, many patients expressed a preference to reduce their cholesterol with lifestyle changes, which could represent a promising area to target in such multifaceted interventions.

Only about 1% of participants reported that they took fewer statins because they had trouble paying for them. Other studies suggest that the adherence to statins is generally higher when patients are prescribed formulary preferred vs. non-preferred statins, or when copayments are lower. [5, 29, 30] Thus, the frequency of cost-related non-adherence could be higher in other settings with greater use of brand statins or where patients face higher out-of-pocket costs. For example, within the same health system among a similar population of adults receiving cardiovascular medications, out-of-pocket costs for statins, diabetes, and hypertension drugs, were generally less than $20 per prescription.[31]

Importantly, our study was conducted prior to the release of the revised ACC-AHA guidelines and others, such as the 2016 United States Preventive States Task Force (USPSTF) recommendations for statin therapy.[32] Thus, our study may not generalize to individuals who are newly recommended to receive statins, such as those without elevated LDL-c levels. In addition, there have been some changes in the drugs available to treat high cholesterol since we conducted the survey, including the availability of generic Lipitor and the FDA approval of proprotein convertase subtilisin/kexin type (PCSK9) inhibitors. We were not able to calculate actual 10-year ASCVD risk levels using the ACC-AHA risk calculator due to missing information for inputs to compare with perceived risk of heart attack. This study was also conducted within an integrated delivery system and adherence levels and communication with providers could be lower in other settings.[6, 7, 33] Lastly, this was a cross-sectional survey and we are not able to assess the causal relationship between actual and perceived risks and adherence.

In conclusion, we found that patients expressed a range of concerns about statins, especially related to lifestyle preferences and the risks of taking statins. The most common reason for reducing statin use, however, was forgetfulness. Although the concerns about statins did not vary substantially across clinical risk categories, patients with lower perceived risk were less likely to adhere to statins, independent of ASCVD diagnoses. Understanding individual barriers and adherence behaviors is critical for designing effective interventions to improve adherence. Focusing on interventions that address both the logistical and informational barriers to statin adherence as well as tailoring interventions by risk level and other patient attitudes could improve the effectiveness of such efforts.

## Supporting information

S1 Questionnaire Survey instrument. This supporting file includes the full survey instrument for the study.
(PDF)
