# Supplementary material for: Patient-reported adherence to statin therapy, barriers to adherence, and perceptions of cardiovascular risk
Source: PLoS One. 2018 Feb 8;13(2):e0191817. doi: 10.1371/journal.pone.0191817 (PMC5805247; doi:10.1371/journal.pone.0191817)
Supplement: S1 Questionnaire — This supporting file includes the full survey instrument for the study. (PDF) [file pone.0191817.s001.pdf]

CARES STUDY

KAISER PERMANENTE®

STUDY ID: ______________________ Date: ___/___/___ Interviewer: _______________________

First, we would like to ask you a question to see if you qualify for this study. All of your answers are confidential.

1. Statin medications are prescription drugs you take for high cholesterol or to prevent high cholesterol. In the last 12 months, were you prescribed or did you have any available prescription refills for statin medications?

[1]☐ Yes → **If Yes**: **Thank you, we have confirmed that you qualify for this study**. *[Continue to Question 2]*

[0]☐ No → **If No,** then: Just to be sure, we would like to confirm that you were not prescribed or had any available refills for the following medications:

- Simvastatin or Zocor,
- Lovastatin or Mevacor,
- or Atorvastatin or Lipitor

→ **If still No, then: Thank you for answering this question. You are not eligible for this study. Thank you very much for your time and cooperation. Have a great day!**

[If respondent was prescribed a statin more than 12 months ago and is unsure whether they have any available refills, say: "Do you think your doctor wants you to continue taking statins?"]

[If respondent is unsure if they have been prescribed a statin medication, offer to give them some examples.]

→ Here are some common examples of statin medications. Please let me know if you have been prescribed any of these:

- Simvastatin or Zocor,
- Lovastatin or Mevacor, or
- Atorvastatin or Lipitor

[If the respondent is still unsure if they have prescribed a statin medication, read additional examples.]

→ Here are some additional examples of statin medications. Please let me know if you have been prescribed any of these:

- Vytorin (which is a combination of simvastatin and ezetimibe)
- Pravastatin or Pravachol
- Rosuvastatin or Crestor, and
- Fluvastatin or Lescol

[If the respondent is still unsure if they have been prescribed a statin medication, ask:]

→ Is there a medication you take that you wonder might be a statin? I can check the name of the drug against a list of statin medications to double check.

- Advicor (niacin extended-release/ lovastatin)
- Altoprev (lovastatin extended-release)
- Caduet (amlodipine and atorvastatin)
- Crestor (rosuvastatin)
- Lescol (fluvastatin)
- Lescol XL (fluvastatin extended-release)
- Lipitor (atorvastatin)
- Mevacor (lovastatin)
- Pravachol (pravastatin)
- Simcor (niacin extended-release/ simvastatin)
- Vytorin (ezetimibe/simvastatin)
- Zocor (simvastatin)

→ *[If the respondent is still unsure if they have prescribed a statin medication, then:]* **Thank you for answering this question. You are not eligible for this study. Thank you very much for your time and cooperation. Have a great day!**

©2010

[If respondent qualifies for study:] **The interview will take approximately 15 to 20 minutes to complete and we will mail you a gift card to thank you for your time. Do you have time now?**

[If Yes, then continue]

It is your choice to participate or not. If you don't want to participate, it will not affect you or your family's care in any way. If you do participate, you do not have to answer any questions you don't want to. All of your answers will be kept confidential and will be used only to come up with averages. The information will NOT go to your doctor, to the health plan, or into your medical record.

If you do not know the answer to a question, please give us your best estimate or guess. Do you have any questions before we start?

2. Why do you think your doctor prescribed a statin medication to you?

____

____

The following questions are about care you received in the last 12 months. Please tell us about care you received both inside and outside of the Kaiser system.

3. a. In the last 12 months, how many different prescription medications did you take?

   0☐ None → **If None, SKIP to Question 4**
   1☐ 1
   2☐ 2-4
   3☐ 5 or more

   b. How many times did you fill your statin prescription at pharmacies outside of Kaiser? ________

   → **If one or more,** c. Which non-Kaiser pharmacy did you use? ________________________

4. On average, how much do you pay for a one-month supply of your statin medication? $________

5. a. In the last 12 months, did you have any non-Kaiser prescription drug coverage from any source, including your partner, spouse, the government (such as Medicaid, or Veterans Affairs), or private insurance?

   0☐ No
   1☐ Yes → **If Yes**, b. Did this insurance cover any of your statin prescriptions? 0☐ No 1☐ Yes

Just to remind you, all of your answers are confidential and will NOT go to your doctor or into your medical record.

6. Please tell us how much you agree with the following statements:	Strongly Agree 1	Agree 2	Disagree 3	Strongly Disagree 4
a. My doctor adequately explained why I was prescribed statin medications and their health benefits	☐	☐	☐	☐
b. My doctor adequately described the risks of taking statin medications	☐	☐	☐	☐
c. My doctor adequately described how I should take my statin medication	☐	☐	☐	☐
d. There was enough time during visits to discuss my statin medication with my doctor	☐	☐	☐	☐
e. I was able to follow up with my doctor about any additional questions I have about my statin medication *[If they did not have any questions, "Do you think you would have been able to follow up…"]*	☐	☐	☐	☐
f. The pharmacist adequately explained why I was prescribed statin medications and their health benefits *["Did you get enough information from your pharmacist about why you were prescribed statin medications…"]*	☐	☐	☐	☐

		Strongly Agree	Agree	Disagree	Strongly Disagree
g.	The pharmacist adequately described the risks of taking statin medications	☐	☐	☐	☐
h.	The pharmacist adequately described how I should take my statin medication	☐	☐	☐	☐

7. Did you do any of the following to find out more information about your statin medication? *[Check all that apply]*

[a]☐ Read the materials provided with your statin prescription from the pharmacy________________________

[b]☐ Talk with a family member or friend____

[c]☐ Look up information in books about health____

[d]☐ Look up information on the internet____

[e]☐ Find out information from television programs, magazines, or newspapers__________________________

[f]☐ Do anything else → g. Please describe____

→ h. *[If respondent answered yes to any of the above, for each specific behavior ask them to describe what kind of information they learned and how helpful it was]*

____

____

Please remember that all of your answers are confidential and will NOT go to your doctor, to the health plan, or into your medical record.

8. a. In the last 12 months, did you decide not to fill a new statin medication prescription?

[0]☐ No
[1]☐ Yes → **If Yes**, b. Did you tell your doctor about this change? [0]☐ No [1]☐ Yes

9. a. In the last 12 months, did you split or skip your statin pills (for example, break them in half or delay refilling your prescription) without your doctor's advice?

[0]☐ No
[1]☐ Yes → **If Yes**, b. Did you tell your doctor about this change? [0]☐ No [1]☐ Yes

10. a. In the last 12 months, did you stop refilling your statin prescription altogether?

[0]☐ No
[1]☐ Yes → **If Yes**, b. In which month did this occur? _______

c. Did you tell your doctor about this change? [0]☐ No [1]☐ Yes

d. Did your doctor tell you to stop taking statins? [0]☐ No [1]☐ Yes

e. Please describe: ____

____

11. On average, in the past month, about how much of your statin medication did you take?

[0]☐ None
[1]☐ 1-24% of the full amount prescribed
[2]☐ 25-49%
[3]☐ 50-79%
[4]☐ 80% or more of the full amount prescribed

(For None through 50-79%:) *[if 8-10 are ALL No, then confirm:]* So, just to reconfirm: *[ask 8-10 again]*

12. a. Besides your statin medication, in the last 12 months did you use any other medication (for any condition) less often than the doctor prescribed (e.g., not filling a prescription, or splitting or skipping pills)?

[0]☐ No
[1]☐ Yes → **If Yes**, b. Which medication(s) did you use less of? ______________________________________

c. Why did you choose to use less of this medication?_________________________________

Next, we would like to ask you about your concerns or experiences with statin medications.

	No 0	Yes 1
13. a. Are you unsure about <u>why you were prescribed a statin medication</u>?	☐	☐
→ **If Yes**, b. Please describe: ______________________________		
c. Because of this, did you take less statin medication than you were prescribed (e.g., not filling a prescription, or splitting or skipping pills)?	☐	☐
14. a. Are you unsure if statin medications <u>lower your cholesterol or your risk of heart disease</u>?	☐	☐
→ **If Yes**, b. Please describe: ______________________________		
c. Because of this, did you take less statin medication than you were prescribed (e.g., not filling a prescription, or splitting or skipping pills)?	☐	☐
15. a. Do you have any <u>trouble paying</u> for your statin medication?	☐	☐
→ **If Yes**, b. Please describe: ______________________________		
c. Because of this, did you take less statin medication than you were prescribed (e.g., not filling a prescription, or splitting or skipping pills)?	☐	☐
16. a. Do you find it <u>inconvenient to obtain</u> your statin medication, such as filling it at the pharmacy or renewing prescriptions?	☐	☐
→ **If Yes**, b. Please describe: ______________________________		
c. Because of this, did you take less statin medication than you were prescribed (e.g., not filling a prescription, or splitting or skipping pills)?	☐	☐
17. a. Do you have <u>trouble remembering</u> to take your statin medication?	☐	☐
→ **If Yes**, b. Please describe: ______________________________		
c. Because of this, did you take less statin medication than you were prescribed (e.g. not filling a prescription, or splitting or skipping pills)?	☐	☐
18. a. Do you find it too <u>difficult or complicated</u> to take your statin medication, for example you are unsure of how often or when to take them or whether you can drink grapefruit juice?	☐	☐
→ **If Yes**, b. Please describe: ______________________________		
c. Because of this, did you take less statin medication than you were prescribed (e.g. not filling a prescription, or splitting or skipping pills)?	☐	☐
19. a. Do you think the potential <u>risks of taking your statin medication outweigh the benefits</u>? *[Read this question slowly and emphasize the words <u>risks</u> and <u>benefits</u>. Repeat the question if respondent doesn't seem to understand]*	☐	☐
→ **If Yes**, b. Please describe: ______________________________		
c. Because of this, did you take less statin medication than you were prescribed (e.g. not filling a prescription, or splitting or skipping pills)?	☐	☐

[If a respondent asks if they should be concerned about these side effects, say: "Serious side-effects from Statins are rare. We are just curious about your general perceptions and any concerns you may have had about these side effects." If respondent is still concerned, repeat the first sentence and add "If you have any additional concerns, don't hesitate to talk to your doctor about them--we're not clinicians and aren't qualified to give specific medical advice."]

No (0) Yes (1)

20. a. Are you concerned about muscle problems, such as soreness, from taking statin medications? ☐ ☐

→ **If Yes**, b. Please describe: ____________________

c. Because of this, did you take less statin medication than you were prescribed (e.g., not filling a prescription, or splitting or skipping pills)? ☐ ☐

d. How likely do you think it is that you will experience this side effect?

- 0 ☐ Not at all likely
- 1 ☐ Very unlikely
- 2 ☐ Somewhat likely
- 3 ☐ Extremely likely
- 4 ☐ I have experienced this side effect

21. a. Are you concerned about liver or kidney problems from taking statin medications? ☐ ☐

→ **If Yes**, b. Please describe: ____________________

c. Because of this, did you take less statin medication than you were prescribed (e.g., not filling a prescription, or splitting or skipping pills)? ☐ ☐

d. How likely do you think it is that you will experience this side effect?

- 0 ☐ Not at all likely
- 1 ☐ Very unlikely
- 2 ☐ Somewhat likely
- 3 ☐ Extremely likely
- 4 ☐ I have experienced this side effect

22. a. Are you concerned about any other side effects from taking statin medications? ☐ ☐

→ **If Yes**, b. Please describe: ____________________

c. Because of this, did you take less statin medication than you were prescribed (e.g., not filling a prescription, or splitting or skipping pills)? ☐ ☐

d. How likely do you think it is that you will experience this side effect?

- 0 ☐ Not at all likely
- 1 ☐ Very unlikely
- 2 ☐ Somewhat likely
- 3 ☐ Extremely likely
- 4 ☐ I have experienced this side effect

23. a. Are you concerned about harmful interactions between statins and other medications you are taking? ☐ ☐

→ **If Yes**, b. Please describe: ____________________

c. Because of this, did you take less statin medication than you were prescribed (e.g., not filling a prescription, or splitting or skipping pills)? ☐ ☐

d. Have you experienced any harmful interactions between statins and other medications you are taking? ☐ ☐

24. a. Are you concerned about having negative long-term health effects from taking your statin medication? ☐ ☐

→ **If Yes**, b. Please describe: ____________________

c. Because of this, did you take less statin medication than you were prescribed (e.g., not filling a prescription, or splitting or skipping pills)? ☐ ☐

	No (0)	Yes (1)
25. a. Are you concerned about having to take a statin medication <u>for the rest of your life</u>?	☐	☐
→ **If Yes**, b. Please describe: ______________________		
c. Because of this, did you take less statin medication than you were prescribed?	☐	☐
26. a. Do you <u>dislike taking medications in general</u>?	☐	☐
→ **If Yes**, b. Please describe: ______________________		
c. Because of this, did you take less statin medication than you were prescribed (e.g., not filling a prescription, or splitting or skipping pills)?	☐	☐
27. a. Would you <u>prefer to lower your cholesterol with lifestyle changes</u> (e.g., exercise, diet) instead of taking statin medications?	☐	☐
→ **If Yes**, b. Please describe: ______________________		
c. Because of this, did you take less statin medication than you were prescribed (e.g., not filling a prescription, or splitting or skipping pills)?	☐	☐
28. a. Are there <u>any other reasons</u> you may have used less of your statin medication than you were prescribed?	☐	☐
→ **If Yes**, b. Please describe: ______________________		

29. Please tell us if any of the following might help you take your statin medication regularly: *[If they ask how often reminders would occur, say: "we're not asking about a specific program, only about how helpful this would be in general." If they are fully adherent, say: "Even though you already take all of your statin medication, would any of the following help you continue to take your statins:]*

	Not at all Helpful (1)	A Little Helpful (2)	Helpful (3)	Very Helpful (4)
a. More <u>time with a doctor</u> to talk about the health benefits, side effects, or risks of statin medications	☐	☐	☐	☐
b. More <u>time with a nurse, pharmacist, or health educator</u> to talk about the health benefits, side effects, or risks of statin medications	☐	☐	☐	☐
c. <u>Written materials</u> describing the health benefits, side effects, and how to take statin medications (e.g., handouts)	☐	☐	☐	☐
d. <u>Information on the Kaiser website</u> describing the health benefits, side effects, and how to take statins	☐	☐	☐	☐
e. <u>In-person classes or workshops</u> that provide information on statin medications	☐	☐	☐	☐
f. <u>Online classes or workshops</u> that provide information on statin medications	☐	☐	☐	☐
g. <u>Phone reminders</u> to take your statin medication or refill a prescription	☐	☐	☐	☐
h. <u>Email reminders</u> to take your statin medication or refill a prescription	☐	☐	☐	☐
i. <u>Postcard reminders</u> to take your statin medication or refill a prescription	☐	☐	☐	☐
j. <u>Text message reminders</u> to take your statin medication or refill a prescription	☐	☐	☐	☐

k. Please describe <u>any other suggestions</u> you have for what might help you take your statin medication regularly:

____

____

Please remember that all of your answers are confidential. If you do not know the answer to a question, please give us your best estimate or guess.

30. Please tell us the effect you think the following factors will have on a person's risk for a heart attack in the next 10 years:

	Lowers the Risk a Lot 1	Lowers the Risk a Little 2	Does Not Change The Risk 3	Raises the Risk a Little 4	Raises the Risk a Lot 5
a. How does taking statins every day affect your risk for a heart attack in the next 10 years? *["If someone takes statins every day, how will this affect their risk for a heart attack in the next 10 years?"]*	☐	☐	☐	☐	☐
b. How does having a family history of heart disease affect the risk for a heart attack in the next 10 years? *["If someone has a history of heart disease, how will this affect their risk for a heart attack in the next 10 years?"]*	☐	☐	☐	☐	☐
c. How does having high cholesterol affect your risk for a heart attack in the next 10 years?	☐	☐	☐	☐	☐
d. How does having a healthy diet affect your risk for a heart attack in the next 10 years?	☐	☐	☐	☐	☐
e. How does regular exercise affect your risk for a heart attack in the next 10 years?	☐	☐	☐	☐	☐
f. How does having diabetes affect your risk for a heart attack in the next 10 years? *[Please answer even if you do not have diabetes yourself]*	☐	☐	☐	☐	☐

31. How likely do you think it is that you will have a heart attack in the next 10 years?

Please tell us a number from 0 to 10, where "0" means that you think there is absolutely no chance, and "10" means that you think it is absolutely sure to happen ("5" means you think there's an equal chance of it happening or not).

[If respondent is hesitant to answer, remind them that this will not affect their care at Kaiser in any way and their answers will be kept completely confidential. You may also add "We know that no one can predict the future. We are just wondering about your perception or best guess.]

Absolutely No Chance ↓ 0   1   2   3   4   5   6   7   8   9   10 ↓ Absolutely Certain

32. Please tell me how much you agree with the following statements:

	Strongly Agree 1	Agree 2	Disagree 3	Strongly Disagree 4
a. I am satisfied overall with Kaiser	☐	☐	☐	☐
b. I am satisfied overall with my regular provider (doctor or nurse practitioner)	☐	☐	☐	☐
c. I trust my doctor's judgments about my medical care	☐	☐	☐	☐
d. I am confident filling out medical forms by myself	☐	☐	☐	☐

The following questions are to help make sure that this study includes many different opinions and groups of people. Please remember that all of your answers are confidential.

33. In general, how would you rate your overall health?

Excellent	Very Good	Good	Fair	Poor	Very Poor
1☐	2☐	3☐	4☐	5☐	6☐

34. a. Have you ever regularly smoked cigarettes (that is, smoked daily for at least a year)?

0☐ No
1☐ Yes → **If Yes**, b. Do you smoke cigarettes now, even occasionally? 0☐No 1☐Yes

35. Are you currently doing any of the following to improve or maintain your health? *[Check all that apply]*

a☐ Getting moderate or vigorous exercise
b☐ Trying to quit smoking or stay off cigarettes
c☐ Eating mostly healthy foods
d☐ Trying to lose weight by exercising and/or dieting
e☐ Trying to manage stress effectively
f☐ Getting enough sleep to feel well-rested
g☐ Limiting alcohol to 1-2 drinks daily or don't drink
h☐ Getting recommended health screenings, tests, or vaccinations

36. Do you have a personal doctor or nurse practitioner at Kaiser? 0☐ No 1☐ Yes

37. What is your marital status? *[Mark only one]*

1☐ Never Married
2☐ Married
3☐ Living with someone as a couple, but not married
4☐ Separated
5☐ Divorced
6☐ Widowed

38. Please indicate the racial/ethnic group(s) that best describes you. Identify all that apply- you can choose more than one. *[If you choose more than one category, please also circle the group with which you most identify]*

a☐ White
b☐ Black or African American
c☐ Hispanic or Latino
d☐ Asian
e☐ Native Hawaiian or Pacific Islander
f☐ American Indian/Alaskan Native
g☐ Other → h. Please describe: ________________________________

39. What is the highest level of school that you have completed?

1☐ 8th grade or less
2☐ Some High School, but did not Graduate
3☐ 12th grade / High School Graduate or Equivalent
4☐ Some College or Two-year Degree
5☐ College Graduate - Four-year Degree (B.A., B.S.)
6☐ Some Graduate School or More

40. a. What was your total household income before taxes during the year 2009? Include money from all sources.

1☐ Less than $20,000
2☐ $20,000 - $29,000
3☐ $30,000 - $39,000
4☐ $40,000 - $59,000
5☐ $60,000 - $99,000
6☐ $100,000 or more

b. How many people, including yourself, did that income support in 2009? ______person(s)

THANK YOU FOR YOUR VALUABLE TIME AND ASSISTANCE!
